# Incidence of human rabies exposure and associated factors at the Gondar Health Center, Ethiopia: a three-year retrospective study

**DOI:** 10.1186/2049-9957-4-3

**Published:** 2015-02-02

**Authors:** Meseret Yibrah, Debasu Damtie

**Affiliations:** School of Biomedical and Laboratory Sciences, College of Medicine and Health Sciences, University of Gondar, Gondar, Ethiopia

**Keywords:** Exposure, Incidence, Rabies, Risk factors

## Abstract

**Background:**

Rabies is one of the oldest known and most feared human diseases. Epidemiological studies provide basic information about the burden of the disease and underline the importance of prevention and control interventions. However, there have been limited studies conducted regarding the incidence of rabies and associated factors in Ethiopia, in general, and in this study area, in particular. Therefore, the aim of this study was to assess the incidence of human rabies exposure and associated factors at the Gondar Health Center, Ethiopia.

**Methods:**

A retrospective cross-sectional study was conducted at the Gondar Health Center where post-exposure prophylaxis (PEP) for rabies was available for the whole population in the North Gondar Zone catchment area. Data of human rabies exposure cases between 2011 and 2013 were collected from the rabies PEP registration book using data abstraction sheets. The data was entered and analyzed using SPSS version 16 statistical software.

**Result:**

A total of 261 cases of human rabies exposure were reported to the Gondar Health Center from 2011 to 2013. The sex and age specific distribution showed that the majority of these cases were among males (142/226, 62.8%) and children under 15 years of age (87/226, 38.5%). A predominant number of cases were observed in individuals from rural areas (161/220, 73.2%), and during fall and winter seasons (67/222, 30.18%). A significant number of people exposed to rabies (23.2%) came to the health center for PEP two or more weeks after the injury. The incidence of human rabies exposure cases was 4.6, 2.61, and 1.27 per 100, 000 population in 2011, 2012, and 2013, respectively. Being male and living in an urban setting were found to be risk factors for human rabies exposure in 2011.

**Conclusion:**

A significant number of human rabies exposure cases were reported to the Gondar Health Center. Being male and living in an urban setting were found to be associated with rabies exposure. A community-based follow-up study is recommended to more accurately estimate the incidence of human rabies exposure.

**Electronic supplementary material:**

The online version of this article (doi:10.1186/2049-9957-4-3) contains supplementary material, which is available to authorized users.

## Multilingual abstracts

Please see Additional file 1 for translations of the abstract into the six official working languages of the United Nations.

## Background

Rabies is a very dangerous viral zoonosis, and all mammals can be infected by the virus. The cause of rabies is a neurotropic virus belonging to the class *Mononegavirales*, the family *Rhabdoviridae*, and the genus Lyssa virus [1]. This disease is endemic in tropical areas and more cases are observed in developing countries [2]. Almost 99.9% of mortalities caused by human rabies as well as 98.5% of animal bite cases occur in tropical areas [3]. According to a World Health Organization (WHO) report, worldwide human deaths from endemic canine rabies are estimated to be 55,000 per annum [4].

Domestic dogs are most important sources of infection with more than 94% of human cases occurring due to a bite from a rabid dog [5]. Rabies has the highest case fatality rate of any conventional infectious disease, approaching the 100% mark. The likelihood of a productive rabies infection after exposure to the virus depends on the dose, route of exposure, site of exposure, variant, host genetic makeup, pre- and/or post-exposure prophylaxis (PEP), etc. [6]. All mammals are susceptible to the Lyssa virus infection, but species-level variation in susceptibility has long been recognized [7].

In Africa, rabies constitutes a serious public health problem [8–10]. In Ethiopia, it is an important disease that has been around for many centuries. Recommended treatments for people bitten by rabid animals, mainly dogs, have been recorded in many Ethiopian medical books since the early 17th century [11]. Rabies fulfills the WHO criteria required to target a certain disease for control [12]. Unlike many other emerging zoonosis, safe and effective animal and human vaccines are widely available for its prevention and control. Despite this, rabies remains a neglected disease that is poorly controlled throughout much of the developing world, particularly in Africa and Asia, where most deaths from human rabies occur [12, 13].

Epidemiological studies provide basic information about the burden of the disease and underline the importance of prevention and control interventions. Therefore, this study was aimed to assess the incidence of human rabies exposure cases and associated factors at the Gondar Health Center in Ethiopia.

## Methods

**Figure 1:**
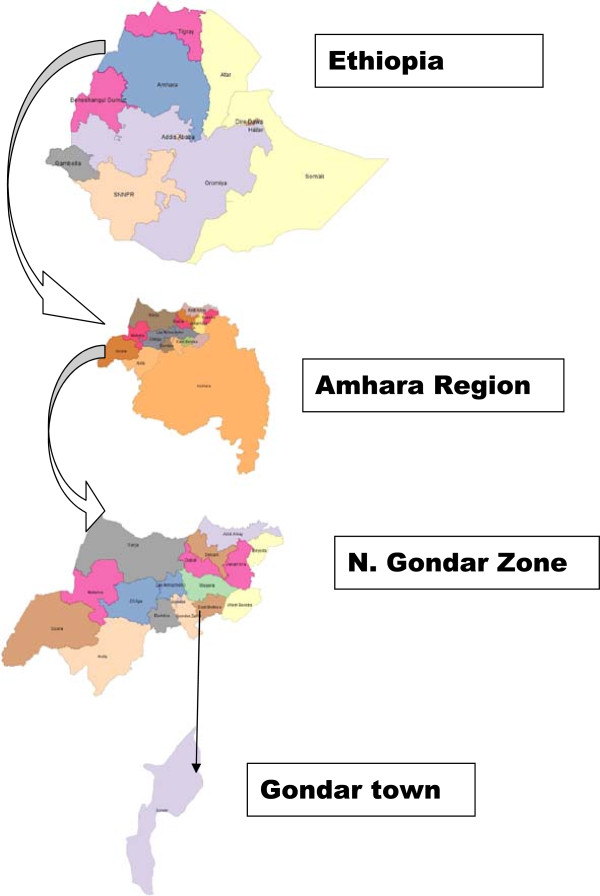
**Map of the study area.**

The study was conducted at the Gondar Health Center, Gondar, Ethiopia (see Figure 1). The city of Gondar is located in the North Gondar Zone of the Amhara Region and is 748 kilometers from the capital, Addis Ababa. The town has a latitude and longitude of 12°36′N 37°28′E, with an elevation of 2,133 meters above sea level. The Gondar Health Center is the only health center in the North Gondar Zone where post-exposure prophylaxis (PPE) for rabies is given.

A retrospective cross-sectional study was conducted to assess the incidence of human rabies exposure and associated risk factors from 2011 to 2013. All people who were exposed to rabies and visited the Gondar Health Center for PEP between 2011 and 2013 made up the study population. Age, sex, season of exposure, residence, and whether the dog survived were the independent variables of the study, while human rabies exposure was treated as an outcome variable.

Data of human rabies exposure cases between 2011 and 2013 were collected from the rabies PEP registration book using data abstraction sheets. The data was entered and analyzed using SPSS version 16 statistical software. A descriptive analysis was performed for categorical variables. Bivariate analysis was performed to examine possible risk factors of human rabies exposure. In all cases, P-values less than 0.05 were considered statistically significant.

Ethical approval was obtained from the School of Biomedical and Laboratory Sciences Research and Ethical Review Committee, College of Medicine and Health Sciences, University of Gondar. Moreover, an official letter was sent to the Gondar Health Center to ensure the study was conducted smoothly.

## Results

A total of 261 human rabies exposure cases were reported to the Gondar Health Center from 2011 to 2013. It was observed that the distribution of human rabies exposure cases year to year decreased: 140, 81, and 40 human rabies exposure cases were reported in 2011, 2012, and 2013, respectively.

The sex specific distribution showed that the majority of rabies exposure cases were among males (142/226, 62.8%). Similarly, children under 15 years of age were the most affected (84/226, 38.5%). This study showed that more rabies exposure cases (161/220, 73.2%) were observed in people from rural areas. In contrast, human rabies exposure in people from urban areas was low (59/220, 26.8%). A significant number of rabies exposure cases were reported during fall and winter (67/222, 30.18%). In contrast, a lower proportion of rabies exposure cases was reported during spring and summer (44/222, 19.8%) (see Table 1).

**Table 1:** **Socio-demographic and seasonal distribution of human rabies exposure cases between 2011 and 2013 at the Gondar Health Center**

Age group	Sex			Residence			Season				
	Male No. (%)	Female No. (%)	Total No. (%)	Urban No. (%)	Rural No. (%)	Total No. (%)	Fall No. (%)	Winter No. (%)	Spring No. (%)	Summer No. (%)	Total No. (%)
<5	10 (7.0)	8 (9.5)	18 (8)	5 (2.3)	13 (5.9)	18 (8.2)	7 (3.2)	3 (1.4)	4 (1.8)	4 (1.8)	18 (8.1)
5–14	43 (30.3)	26 (31)	69 (30.5)	12 (5.5)	53 (24.1)	65 (29.5)	13 (5.9)	22 (9.9)	15 (6.8)	19 (8.6)	69 (31.1)
15–24	25 (17.6)	19 (22.6)	44 (19.5)	15 (6.8)	29 (13.2)	44 (20)	15 (6.8)	11 (5.0)	7 (3.2)	9 (4.1)	42 (18.9)
25–34	26 (18.3)	11 (13.1)	37 (16.4)	11 (5)	25 (11.4)	36 (16.4)	12 (5.4)	15 (6.8)	7 (3.2)	2 (0.9)	36 (16.2)
35–44	15 (10.6)	4 (4.8)	19 (8.4)	6 (2.7)	13 (5.9)	19 (8.6)	6 (2.7)	6 (2.7)	4 (1.8)	3 (1.4)	19 (8.6)
45–54	10 (7)	9 (10.7)	19 (8.4)	3 (1.4)	15 (6.8)	18 (8.2)	6 (2.7)	6 (2.7)	3 (1.4)	3 (1.4)	18 (8.1)
55–64	5 (3.5)	3 (3.6)	8 (3.5)	3 (1.4)	5 (2.3)	8 (3.7)	2 (0.9)	2 (0.9)	2 (0.9)	2 (0.9)	8 (3.6)
>/=65	8 (5.6)	4 (4.8)	12 (5.3)	4 (1.8)	8 (3.6)	12 (5.4)	6 (2.7)	2 (0.9)	2 (0.9)	2 (0.9)	12 (5.4)
**Total**	142 (62.8)	84 (37.2)	226 (100)	59 (26.8)	161 (73.2)	220 (100)	67 (30.2)	67 (30.2)	44 (19.8)	44 (19.8)	222 (100)

**Figure 2:**
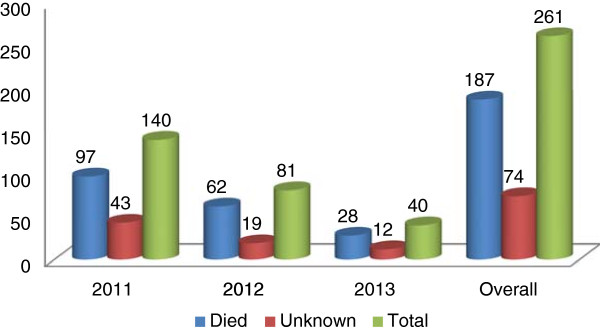
**Dog survival status at the time of victim’s visit to the Gondar Health Center for PEP, from 2011 to 2013.**

The time of arrival to the health center after rabies exposure was variable. Most of the people exposed to rabies visited the center within a week, or between one and two weeks after exposure. However, a significant number of people (24.7%) exposed to rabies came to the health center for PEP two or more weeks after the injury (see Table 2). All human rabies exposure cases were due to a bite from a dog. The majority of the dogs died by the time of the visit to the health center (71.65%) and the survival statuses of the rest were unknown (see Figure 2).

**Table 2:** **Time of arrival to the Gondar Health Center for PEP after human rabies exposure from 2011 to 2013**

Variables		Immediately No. (%)	Within a week No. (%)	Between 1–2 weeks No. (%)	Between 2–4 weeks No. (%)	>4 weeks No. (%)
**Sex**	Male	27 (16.9)	44 (27.5)	46 (28.8)	29 (18.1)	14 (8.8)
	Female	16 (17.6)	32 (35.2)	24 (26.4)	14 (15.4)	5 (5.5)
	**Total**	**43 (17.1)**	**76 (30.3)**	**70 (27.9)**	**43 (17.1)**	**19 (7.6)**
**Residence**	Urban	13 (22.0)	19 (32.2)	15 (25.4)	10 (16.9)	2 (3.4)
	Rural	30 (16.9)	54 (30.3)	51 (28.7)	27 (15.2)	16 (9.0)
	**Total**	**43 (18.1)**	**73 (30.8)**	**66 (27.8)**	**37 (15.6)**	**18 (7.6)**

The incidence of human rabies exposure cases as it was calculated from the mid-year population was 4.6, 2.61, and 1.27 per 100,000 population for the three respective study years. In all three years in which the study took place, males were more exposed to rabies (see Table 3). Being male and living in an urban setting (see Table 4) were found to be risk factors for human rabies exposure from bivariate analysis [COR = 1.5967 (95% CI = 1.1347–2.2467) and COR = 2.6328 (95% CI = 1.8092–3.8311)], respectively, for the year 2011.

**Table 3:** **Incidence of human rabies exposure cases in the North Gondar Administrative Zone by sex from 2011 to 2013**

Year	Sex	Population	Reported cases	Incidence per 100,000 population	COR (95% CI)
**2011**	Male	1,542,518	87	5.64	1.5967 (1.1347–2.2467)*
	Female	1,500,347	53	3.53	1
	Total	3,042,865	140	4.6	
**2012**	Male	1,571,760	52	3.30	1.4752 (1.1081–2.7488)*
	Female	1,529,784	29	1.90	1
	Total	3,101,544	81	2.61	
**2013**	Male	1,601,699	25	1.56	1.6232 (0.8558–3.0789)
	Female	1,559,956	15	0.96	1
	Total	3,161,655	40	1.27	

COR = crude odds ratio, CI = confidence interval, *statistically significant association.

**Table 4:** **Incidence of human rabies exposure cases in the North Gondar Administrative Zone by residence from 2011 to 2013**

Year	Sex	Population	Reported cases	Incidence per 100,000 population	COR (95% CI)
**2011**	Urban	456,879	40	8.76	2.6328 (1.8092–3.8311)*
	Rural	2,585,986	86	3.32	1
	Total	3,042,865	126	4.14	
**2012**	Urban	476,476	13	2.73	1.0533 (0.5819–1.9064)
	Rural	2,625,068	68	2.59	1
	Total	3,101,544	81	2.61	
**2013**	Urban	496,914	10	2.01	1.8492 (0.9012–3.7944)
	Rural	2,664,741	29	1.09	1
	Total	3,161,655	39	1.23	

COR = crude odds ratio, CI = confidence interval, *statistically significant association.

## Discussion

A significant number of human rabies exposure cases (261) were reported to the Gondar Health Center from 2011 to 2013. The sex specific distribution showed that the majority (62.8%) of the rabies cases were among males. This finding was supported by a previous Ethiopian study and a Nigerian study [14, 15]. This might be explained by the activities males are frequently involved in: they might do more nightly and outdoor activities while females are more likely to remain indoors due to cultural and religious reasons. A large proportion human rabies exposure cases were reported among children under 15 years of age (38.5%). This finding is in line with studies done in Tanzania and Ethiopia [14, 16]. This could potentially be explained by the fact that children are more likely to provoke dogs and are also less likely to be able to defend themselves, thereby being more exposed to dog bite injuries. Unlike the present study, a study conducted in New York reported no significant differences among sex or age distributions and rabies exposure [17].

This study showed that the majority of the rabies exposure cases (161/220, 73.2%) were observed in people from rural areas, which is concurrent with the study done in New York [17]. This might possibly be explained by the fact that larger populations reside in rural areas in Ethiopia compared to urban settings.

The majority of rabies exposure cases were reported to occur during fall and winter (67/222, 30.18%). Contrary to this finding, studies from New York, Tanzania, and Nigeria reported high incidence of rabies exposure during spring and summer [15–17]. This seasonal variation could be due to variations in geographical locations which determine the weather conditions of each season.

A significant number of people exposed to rabies (24.7%) came to the health center for PEP late, which would hamper the efficacy of the PEP. Unlike our finding, a Nigerian retrospective study revealed that most (87.7%) of the victims afflicted with dog bite injuries presented at a clinic within 24 hours of the bite occurring, which is commendable [15]. The explanation for our study’s finding might be due to the health center’s inaccessibility in terms of distance for the majority of the rural catchment area, as the Gondar Health Centre is the only facility providing PEP.

Dog bite was the only source of rabies exposure in this study. This finding is consistent with another community-based active surveillance study conducted in Kenya [18]. Among dogs responsible for rabies exposure, the majority (71.65%) died at the time of the victim’s visit to the health center and the survival statuses of the rest were unknown. This implies that dogs were the potential sources of the rabies infection.

The incidence of human rabies exposure cases as it was calculated from the mid-year population was 4.6, 2.61, and 1.27 per 100,000 population for the three respective study years. This finding is by far lower than reports from studies in New York (27/100,000), Tanzania (58/100,000), and a Kenyan active surveillance report (234/100,000) [16–18]. These discrepancies might be explained by multiple socio-cultural factors and methodological differences.

Although we didn’t exhaustively assess all possible risk factors for human rabies exposure due to the limited data available in the log book, being male was found to be a risk factor for human rabies exposure for the year 2011.This was supported by studies from Tanzania, Ethiopia, and Nigeria [14–16]. The present study also found that living in an urban setting is a risk factor, unlike the report from New York. This might be due to the fact that in Ethiopia people in rural settings settle very far apart in contrary to urban areas where people live in crowded conditions, which may favor dog bite exposure. As well as that, stray dogs are abundant in urban settings which may contribute to human rabies exposure.

## Conclusion

A significant number of cases of human rabies exposure was reported to the Gondar Health Centre despite the fact that the majority of victims would have preferred traditional healers for post-exposure management of rabies (which may have reduced the actual figure). Dog bite was the only source of exposure reported. A significant number of people exposed to rabies came to the health center to seek medical attention late. Being male and living in an urban setting were found to be the risk factors for rabies exposure in 2011. A community-based follow-up study is recommended to more accurately estimate the incidence of human rabies exposure.

## Electronic supplementary material

Additional file 1: **Multilingual abstracts in the six official working languages of the United Nations.** (PDF 247 KB)

## Abbreviations

OR: Odds ratio
PEP: Post-exposure prophylaxis
WHO: World Health Organization.
