# Supplementary material for: Incidence of human rabies exposure and associated factors at the Gondar Health Center, Ethiopia: a three-year retrospective study
Source: Infect Dis Poverty. 2015 Feb 2;4(1):3. doi: 10.1186/2049-9957-4-3 (PMC4327962; doi:10.1186/2049-9957-4-3)
Supplement: Supplementary file 1 — Additional file 1: Multilingual abstracts in the six official working languages of the United Nations. (PDF 247 KB) [file 40249_2014_92_MOESM1_ESM.pdf]

Translation of the abstract into the six official working languages of the United Nations

حدوث الإصابة بداء الكلب البشري والعوامل المرتبطة بها بمركز جوندار الصحي، إثيوبيا: دراسة بأثر رجعي لمدة ثلاث سنوات

ميسرت يبره، ديباسو دامتي

الملخص

الخلفية: يعتبر داء الكلب من أقدم الأمراض المعروفة والأكثر رعباً للإنسان. وتوفر الدراسات الوبائية معلومات أساسية عن أعباء المرض وتؤكد على أهمية تدخلات الوقاية والمكافحة. ومع ذلك، كانت هناك دراسات محدودة أجريت فيما يتعلق بحدوث داء الكلب والعوامل ذات الصلة في إثيوبيا، بصفة عامة، وفي هذه الدراسة بصفة خاصة.

الهدف: كان الهدف من هذه الدراسة هو تقييم حدوث الإصابة بداء الكلب البشري والعوامل المرتبطة بمركز جوندار الصحي، إثيوبيا.

طرق العمل: تم إجراء دراسة عرضية بأثر رجعي بمركز جوندار الصحي حيث العلاج الوقائي بعد التعرض (PEP) لداء الكلب كان متاحاً لجميع السكان بمنطقة الخدمات الطبية في منطقة شمال جوندار. بيانات حالات التعرض لداء الكلب البشري بين عامي 2011 و 2013 تم جمعها من دفتر تسجيل العلاج الوقائي بعد التعرض لداء الكلب باستخدام كشوف تجريد البيانات. تم إدخال البيانات وتحليلها باستخدام البرنامج الإحصائي SPSS الإصدار 16.

النتائج: تم حضور ما مجموعه 261 حالة تعرض لداء الكلب البشري إلى مركز جوندار الصحي من 2011 إلى 2013. التصنيف الدقيق حسب الجنس والعمر أظهر أن غالبية هذه الحالات كانت بين الذكور (226/142، 62.8 %) والأطفال دون سن 15 عاماً (226/87، 38.5 %). ولوحظ أن العدد الغالب من الحالات هم أفراد من المناطق الريفية (220/161، 73.2 %)، وخلال فصلي الخريف والشتاء (222/68، 30.18 %). وجاء عدد كبير من الأشخاص المصابين بداء الكلب (23.3 %) إلى المركز الصحي للحصول على العلاج الوقائي بعد التعرض بعد أسبوعين أو أكثر من الإصابة. حدوث حالات إصابة بداء الكلب البشري كان 4.6، و 2.61 و 1.27 لكل 100,000 نسمة خلال أعوام 2011، و 2012 و 2013 على التوالي. تم اكتشاف أن الذكور الذين يعيشون في المناطق الحضرية لديهم عوامل خطر تعرضهم للإصابة بداء الكلب البشري في عام 2011.

الاستنتاج: تم حضور عدد كبير من حالات الإصابة بداء الكلب البشري إلى مركز جوندار الصحي. تم اكتشاف أن الذكور الذين يعيشون في المناطق الحضرية لديهم ارتباط بالإصابة بداء الكلب. ينصح بإجراء دراسة متابعة مجتمعية للحصول على تقييم أكثر دقة لحدوث الإصابة بداء الكلب البشري.

Translated from English version into Arabic by Saher Salama, through

TRANSLATORS WITHOUT BORDERS

埃塞俄比亚贡德尔医疗中心的一项为期 3 年的针对人狂犬病暴露的发生率及危险因素的回顾性研究

Meseret Yibrah, Debasu Damtie

摘要

引言：狂犬病是一种年最古老和最可怕的疾病。流行病学研究为该病的疾病负担提供了基本信息，并强调了预防和控制措施的重要性。然而，埃塞俄比亚缺乏对狂犬病的发病率及其影响因素的相关研究。

目的：本研究的目的是评估埃塞俄比亚贡德尔医疗中心人狂犬病暴露于传染源后的发病率及其影响因素。

方法：在贡德尔医疗中心进行了一项回顾性横断面研究，研究对象为北贡德尔区内所有接受狂犬病暴露后预防（PEP）的人群。所用数据为 2011-2013 年狂犬病暴露后预防登记簿中的人狂犬病暴露病例。使用 SPSS 16.0 统计软件进行数据输入和分析。

结果：贡德尔医疗中心 2011-2013 年共上报 261 例人狂犬病暴露病例。性别和年龄分布显示，病例多为男性（142 / 226，62.8%）和 15 岁以下儿童（87 / 226，38.5%）。病例主要发生在农村地区（161/220，73.2%），

并在秋冬季节高发（67 / 222，30.18%）。值得注意的是，23.2%的暴露病例在受伤后 2 周或 2 周以上才到医疗中心接受暴露后预防。在 2011、2012 和 2013 年人狂犬病发病率分别为 4.6/10 万、2.61/10 万和 1.27/10 万，此外，男性和在城市居住是 2011 年人狂犬病暴露的危险因素。
结论：贡德尔医疗中心报告了相当数量的狂犬病暴露病例。男性和城市居住与人暴露于狂犬病传染源相关。建议开展一个以社区为基础的后续研究以更准确估算人狂犬病的发病率。

Translated from English version into Chinese by Zheng Qi, through

CHINESE CENTER FOR DISEASE CONTROL AND PREVENTION
NATIONAL INSTITUTE OF PARASITIC DISEASES

Incidence de l'exposition à la rage humaine et facteurs associés au Centre de Santé de Gondar, Éthiopie : une rétrospective de trois ans d'étude

Meseret Yibrah, Debasu Damtie

Résumé

Information : La rage fait partie des maladies humaines les plus anciennement connues et les plus redoutées. Les études épidémiologiques fournissent des informations basiques sur la charge de la maladie et soulignent l'importance de la prévention et le contrôle des interventions. Cependant, les études menées sont limitées étant donné l'incidence de la rage et des facteurs associés en Éthiopie, en général, et dans le domaine de cette étude, en particulier.
Objectif : Le but de cette étude était d'évaluer l'incidence de l'exposition à la rage humaine et les facteurs associés, au Centre de Santé de Gondar, Éthiopie.
Méthodes : Une étude rétrospective transversale a été conduite au Centre de Santé de Gondar où la prophylaxie post-exposition (PPE) pour la rage était disponible pour toute la population de la zone de la circonscription du nord de Gondar. Des données concernant des cas d'exposition à la rage humaine entre 2011 et 2013 ont été collectées à partir du registre de la PPE en utilisant les feuilles d'abstraction de données. Les données ont été saisies et analysées en utilisant le programme de statistiques SPSS version 16.
Résultats : Un total de 261 cas d'exposition à la rage humaine ont fait l'objet d'un rapport au Centre de Santé de Gondar entre 2011 et 2013. La répartition spécifique suivant le sexe et l'âge a démontré que la majorité de ces cas se trouvait chez des hommes (142/226, 62.8%) et des enfants de moins de quinze ans (87/226, 38.5%). Un nombre prédominant de cas ont été observés chez des individus des zones rurales (161/220, 73.2%), et pendant l'automne et l'hiver (67/222, 30.18%). Un nombre significatif de personnes exposées à la rage (23.2%) sont venues au centre de santé pour la PEP deux semaines ou plus après la blessure. L'incidence de cas d'exposition à la rage humaine était respectivement de 4.6, 2.61, et 1.27 pour 100 000 en 2011, 2012, et 2013. Le fait d'être de sexe masculin et de vivre en milieu urbain se révèlent être les facteurs de risque d'exposition à la rage humaine en 2011.
Conclusion : Un nombre significatif de cas d'exposition à la rage humaine ont été rapportés au Centre de Santé de Gondar. Être de sexe masculin et vivre en milieu urbain se révèlent être les facteurs associés à l'exposition à la rage. Une étude communautaire de suivi est recommandée pour estimer avec plus de précision l'incidence de l'exposition à la rage humaine.

Translated from English version into French by v-mazet, through

Заболеваемость бешенством у человека и сопряженные с этим факторы в медицинском центре Гондар, Эфиопия: Трехгодичное ретроспективное исследование

Месерет Ибра, Дебасу Дамти (Meseret Yibrah, Debasu Damtie)

Синопсис

Справочная информация: Бешенство является одним из старейших известных человечеству заболеваний и одним из наиболее страшных. Эпидемиологические исследования дают основную информацию о заболевании и подчеркивают необходимость мер профилактики и борьбы с ним. Однако, наблюдается недостаток исследований о возникновении бешенства и сопутствующих факторов в Эфиопии вообще и в этой области исследования в частности.

Цель: Целью данного исследования было оценить распространение бешенства среди населения и факторы, сопутствующие заболеванию, в медицинском центре Гондар, Эфиопия.

Методология: Ретроспективное межгрупповое исследование проводилось на базе медицинского центра Гондар, где для всего населения района Северный Гондар предлагалась постконтактная профилактика (ПКП) бешенства. Данные о случаях постконтактного заражения бешенством собирались в период между 2011 и 2013 гг. с использованием ПКП журналов для абстрагирования информации. Данные вводились и анализировались с использованием версии 16 статистического программного обеспечения SPSS.

Результаты: Всего 261 случай заражения бешенством у людей был зарегистрирован в медицинском центре Гондар в период между 2011 и 2013 гг. Специфическое распределение по полу и возрасту показало, что большинство случаев имели место среди мужчин (142/226, 62,8%) и детей младше 15 лет (87/226, 38,5%). Подавляющее большинство случаев были зарегистрирован среди сельского населения (161/220, 73,2%), осенью и зимой (67/222, 30,18%). Значительное количество людей, вступивших в контакт с бешенством (23,2%) прибыли в медицинский центр для ПКП через две или больше недель после возможного инфицирования. Частота случаев бешенства среди людей составила 4,6, 2,61 и 1,27 на 100 000 населения в 2011, 2012 и 2013 гг. соответственно. Факторами риска для человеческого заражения бешенством в 2011 году являлись принадлежность к мужскому полу и проживание в городе.

Заключение: Значительное количество случаев заражения бешенством среди людей было зарегистрировано в медицинском центре Гондар. Принадлежность к мужскому полу и проживание в городе ассоциировались с повышенным риском заражения бешенством. Рекомендуется провести дополнительное исследование среди местного населения для получения более точных данных о заражении бешенством у людей.

Translated from English version into Russian by Julia Zarubinska-Toepritz, through

Incidencia de la exposición a la rabia en humanos y factores relacionados en el centro de salud de Gondar, Etiopía: estudio retrospectivo de tres años

Meseret Yibrah, Debasu Damtie

Resumen

Antecedentes: La rabia es una de las enfermedades humanas más antigua y temida. Los estudios epidemiológicos aportan información básica acerca de la carga de la enfermedad y subrayan la importancia de las intervenciones para su prevención y control. No obstante, se han realizado pocos estudios sobre la incidencia de la rabia y factores relacionados en Etiopía en general, y en este ámbito de estudio en particular.

Objetivos: El objetivo del presente estudio consistía en evaluar la incidencia de la exposición a la rabia en humanos y factores relacionados en el centro de salud de Gondar, Etiopía.

Métodos: Se realizó un estudio transversal en el centro de salud de Gondar donde estaba disponible el tratamiento postexposición PEP para toda la población de la zona de captación del norte de Gondar. Los datos de exposición a la rabia en humanos entre los años 2011 y 2013 se recopilaron del libro de registro de la enfermedad del PEP utilizando hojas de abstracción de datos. Los datos fueron introducidos y analizados con el software estadístico SPSS versión 16.

Resultados: Entre los años 2011 y 2013 se notificaron al centro de salud de Gondar un total de 261 casos de exposición a la rabia en humanos. La distribución específica por género y edad mostró que la mayoría de dichos casos se dieron entre hombres (142/226, el 62,8%) y niños menores de 15 años (87/226, el 38,5%). Un número predominante de casos fueron observados en individuos procedentes de zonas rurales (161/220, el 73,2%) y durante las estaciones de otoño y primavera (67/222, el 30,18%). Un número importante de personas expuestas a la rabia (el 23,2%) llegó al centro de salud para el tratamiento PEP dos semanas o más después de la lesión. La incidencia de los casos de exposición a la rabia en humanos fue de 4,6, 2,61 y 1,27 por cada 100.000 habitantes en los años 2011, 2012 y 2013 respectivamente. Se descubrió que ser hombre y vivir en un entorno urbano suponían factores de riesgo para la exposición a la rabia en humanos en 2011.

Conclusión: Se notificaron al centro de salud de Gondar un número significativo de casos de exposición a la rabia en humanos. Se descubrió que ser hombre y vivir en un entorno urbano suponían factores de riesgo para la exposición a la rabia. Se recomienda un estudio de seguimiento basado en la comunidad para estimar de modo más preciso la incidencia de la exposición a la rabia en humanos.

Translated from English version into Spanish by Raquel Bentué, through

TRANSLATORS
WITHOUT BORDERS
